# The impact of hepatocyte nuclear factor-1α on liver malignancies and cell stemness with metabolic consequences

**DOI:** 10.1186/s13287-019-1438-z

**Published:** 2019-11-04

**Authors:** Xue Wang, Waseem Hassan, Jing Zhao, Sahar Bakht, Yunjuan Nie, Ying Wang, Qingfeng Pang, Zhaohui Huang

**Affiliations:** 1 Laboratory of Cancer Epigenetics, Wuxi School of Medicine, Jiangnan University, Wuxi, China; 2 Department of Pharmacy, COMSATS University Islamabad, Lahore campus, Lahore, Pakistan; 3 Department of Pharmacy, The Islamia University of Bahawalpur, Bahawalpur, Pakistan; 4 Wuxi Cancer Institute, Affiliated Hospital of Jiangnan University, Wuxi, 214062 Jiangsu China; 5 Department of physiopathology, Wuxi School of Medicine, Jiangnan University, Wuxi, Jiangsu province China

**Keywords:** HNF-1α, Liver malignancies, Metabolic repercussions, Stem initiation

## Abstract

Hepatocyte nuclear factor-1 alpha (HNF-1α) is a transcription factor expressed predominantly in the liver among other organs. Structurally, it contains POU-homeodomain that binds to DNA and form proteins that help in maintaining cellular homeostasis, controlling metabolism, and differentiating cell lineages. Scientific research over the period of three decades has reported it as an important player in various liver malignancies such as hepatocellular cancers (HCCs), hepatocellular adenoma (HA), and a more specific HNF-1α-inactivated human hepatocellular adenoma (H-HCAs). Abundant clinical and rodent data have noted the downregulation of HNF-1α in parallel with liver malignancies. It is also interesting to notice that the co-occurrence of mutated HNF-1α expression and hepatic carcinomas transpires typically along with metabolic repercussion. Moreover, scientific data implies that HNF-1α exerts its effects on cell stemness and hence can indirectly impact liver malignancies and metabolic functioning. The effects of HNF-1α on cell stemness present a future opportunity to explore a possible and potential breakthrough. Although the mechanism through which inactivated HNF-1α leads to hepatic malignancies remain largely obscure, several key signal molecules or pathways, including TNF-α, SHP-1, CDH17, SIRT, and MIA-2, have been reported to take part in the regulations of HNF-1α. It can be concluded from the present scientific data that HNF-1α has a great potential to serve as a target for liver malignancies and cell stemness.

## Introduction

Hepatocyte nuclear factors (HNFs) were initially discerned as liver-enriched transcription factors that could play multiple roles in the transcription of liver-specific genes. However, with the passage of time, it became obvious that HNFs are not restricted to the liver only. Since then, it has been identified in the pancreas, kidneys, intestine^,^ spleen, thymus, testis, keratinocytes, and melanocytes of human skin. There are four different structural classes of HNF primarily based on their DNA binding domain (DBD), namely HNF1, HNF3 (FOXA), HNF4, and HNF6 [1]. Among these, HNF1 is most widely recognized due to its relatively wider scope and functionalities. HNF-1 includes HNF-1α (TCF1) and HNF-1β (TCF2) that both further encode three isoforms (A, B, and C). Both HNF-1α and HNF-1β contain a POU-homeodomain that binds to DNA.

HNF-1α is encoded by hepatocyte nuclear factor 1 homeobox A gene located in 12q24.2 and containing 9 exons. HNF-1α contains 3 functional domains, N-terminal dimerization domain (between residues 1 and 32), a bipartite DNA-binding motif having atypical POU-homeodomain (between residues 98 and 280), and a C-terminal transactivation domain (between residues 281 and 631) [2]. The two isoforms can form dimers to regulate transcription of target genes. Unlike other POU transcription factors that bind DNA either as monomers or dimer, HNF-1α and HNF-1β exclusively bind as dimers. Dimerization of HNF-1α and HNF-1β and their respective isoforms collectively adds to the functional complexity and diversity of these proteins. Though these proteins share similar DNA-binding characteristics, their activation domains are distinct. For the same reason, homodimers of HNF-1α and HNF-1β trigger the transcription, while heterodimers of these two proteins have a potential to block HNF-1α-dependent genes as some isoforms of HNF-1β are deficient in activation domain. The plausible explanation behind functional diversity of HNF-1α and HNF-1β in hepatocellular carcinoma can be traced in their structural details. Today, it has been proved that HNF-1α shows protective behavior against cancer, while HNF-1β seems to promote tumorigenesis. Interestingly, monomers of both proteins exchange freely with each other to form homo- and heterodimers. This possibly explains the importance of immediate microenvironment in the liver and other organs that may or may not facilitate the transcriptional relationship between HNF-1α and HNF-1β. DCoH (transcriptional coactivator dimerization cofactor) blocks subunit exchange by associating with the dimerization domain. Sequence homology and NMR have confirmed the coiled-coil or a four-helix bundle structure of HNF-1α and HNF-1β.

HNF-1α performs a variety of important functions predominantly related to cellular homeostasis and metabolism in vital organs through transcription. Thus far, it has been reported to affect cell lineage differentiation, lipid metabolism, glucose metabolism—with wider repercussion on diabetes, angiotensin-converting enzyme 2 (ACE2), pancreatic development, β-cell growth, proteins involved in type II diabetes, bile acid transporters in the kidneys, and drug metabolism.

Although HNF-1α impacts a wide range of organs, the hepatic responses are most pronounced due to its higher concentration and local production. For instance, it showed hints of effectiveness in NAFLD and hepatic fibrosis models in which hepatic knockout of HNF-1α in rats notably worsen liver fibrosis, while re-expression helped alleviation. Interestingly, it was also involved in liver lipid metabolism. For instance, Rufibach et al. systematically proved the regulation of human hepatic lipase gene (LIPC) by HNF-1α and other transcription factors in vitro and in vivo [3]. Correspondingly, Niemann-Pick C1-like 1 (NPC1L1), an important regulator of intestinal cholesterol absorption, is also regulated by HNF-1α [4]. Additionally, HNF-1α could modulate the transcription of a number of hepatic-specific genes encoding CYP2E1, albumin, phosphoenolpyruvate carboxykinase, phenylalanine hydroxylase, α-1 antitrypsin, α- and β-fibrinogen, and clotting factors [5]. Beyond its regulatory role in hepatic lipid and glucose metabolism, HNF-1α has gained a significant reputation as a promising drug target for HCC. Relatedly, genome-wide association study (GWAS) data has linked HNF1A with the increased risk in pancreatic cancers [6]. In a set of in vitro experiments, Luo et al. showed tumor suppressor role of HNF1A in multiple cancer cell lines. The expression of HNF1A was lower in cancer cell lines as compared to non-cancerous cell lines. Furthermore, silencing of HNF1A expression raised the rate of pancreatic cancer cell proliferation [7]. Over the period of three decades, notable HNF-1α data on HCC and related cancers perpetually drizzled over scientific horizon, but these efforts lack proper compilation. According to the best of our knowledge, this is the first review that precisely focuses on the roles of HNF-1α in HCC and cell stemness.

### Mutated HNF-1α causes liver malignancies with metabolic repercussions

Well-known and robust HNF-1α relationship with lipid and glucose metabolism faces greater chances of metabolic repercussions in the backdrop of HNF-1α-inactivation-associated liver cancers. For instance, Patitucci et al. showed that AKT2 phosphorylated and inhibited HNF-1α which alleviated the suppression of hepatic PPARγ expression, resultantly promoting tumorigenesis [8]. Previously, Rebouissou and colleagues have established clinical-based study spanning over a decade including 40 human hepatocellular adenomas (HCAs) that are linked with the inactivated or mutated HNF-1α expression, 25 non-steatotic non-tumor livers, and 11 steatotic non-tumor livers. Anticipatorily, modes of lipogenesis like repression of gluconeogenesis, activation of glycolysis, citrate shuttle, and fatty acid synthesis manifested a parallel presence in these HCAs [9]. Moreover, it was delineated that HNF-1α knockout mice increased fatty acid synthesis in the liver in parallel with the spontaneous development of HCC through fatty liver without cirrhosis [10]. Furthermore, a retrospective study noted 88% of the HNF-1α-mutated HCAs with fatty components and hypovascular pattern having greater sensitivity and specificity [11]. In 2010, the relationship between HNF-1α inactivation and various tumor-promoting and developmental mechanisms was reconfirmed. Congruent with previous results, HNF-1α inactivation also showed induction of glycolysis and raised lipogenesis in parallel with the activation of the mTOR pathway [12]. These results put an indirect light and reassure on much-established notion of lipid-led cancers. Loss of liver fatty acid-binding protein P (L-FABP) is linked with the induction of hepatic lipid accumulation, and its normal expression is associated with preventing age or diet-induced obesity. Loss of L-FABP and HNF-1α inactivation is a consistent feature of fibrolamellar carcinoma while HNF-1α mutation is an important occurrence in fibrolamellar carcinoma pathogenesis [13].

Mutations in HNF-1α are well-acknowledged in diabetes and are frequently mentioned in liver malignancies. For example, HNF-1α knockout mice are characteristically described as the phenotypes of both Laron-type dwarfism and non-insulin-dependent diabetes mellitus (NIDDM) [14]. HNF-1α-inactivated HA is known as a MODY3-related disease due to mutations in HNF-1α. Reznik et al. showed the occurrence of liver adenomatosis in six MODY3-affected patients from two unrelated large families, and a hot-spot germline mutation P291fs of HNF-1α was identified in the two pro-bands and 16 relatives from the two families. Consequently, MODY3-affected patients should be screened for liver adenomatosis as they carry significant risk as a result of HNF-1α mutations [15]. HNF-1α mutations are also linked with increased tissue glucose uptake, which can serve as a worsening factor in carcinomas. Ozaki et al. shed light on the potential mechanism of HNF-1α-inactivated HCAs (H-HCAs), and they found increased glucose uptake owing largely to GLUT2 and HK4 expression and G6PT1 inactivation [16]. Although the exact mechanism linking HNF-1α mutations with the development of diabetes is partially known, there are a number of studies that suspect structural mutations as a plausible explanation. The discussion in this section implicates HNF-1α mutations in accumulating lipids in the liver, promoting diabetes, and raising glucose uptake that can better help survive cancerous cells.

### Human and animal evidences link HNF-1α with liver malignancies

Based on the well-acknowledged fact that HCA is associated with oral contraception, CYP1B1 germ line-inactivating mutations were verified increasing the incidence of HCA in women with HNF-1α mutations [17]. In an attempt to identify HCAs at risk for malignant transformation, Miller et al. identified 34 HNF-1α-inactivated patients out of total 97 HCAs [18]. The repeat implications of HNF-1α mutations with HCAs make it an instinctive suspect in HCCs as well. A point mutation of HNF-1α (c.A1532 > T/p.Q511L) identified in HCC patients promoted proliferation, migration, and invasion in HCC cells [19]. Relatively more comprehensive evidences were provided by Zeng and companions. They isolated cancer cells and tumor-associated fibroblasts (TAFs) from human HCC tissues and proved that HCC cells have blunted expression of HNF-1α, whereas forced re-expression of HNF-1α reduced the in vitro proliferation of HCC and TAF cells. Moreover, the in vivo assays confirmed the anti-tumor effects of HNF-1α using HCC xenograft models in nude mice [20]. The HCC patients with positive HNF-1α expression had a better prognosis. The co-existence of inflammatory H-HCAs has raised eyebrows for more subtle HNF-1α role in causing multiple adenomas. A scientific report seeking to sub-classify HCAs showed prevalence of ~ 30% HNF-1α-inactivated cases, suggesting HNF-1α as a molecular classification maker for HCA [21].

One of the earlier evidences linked poor histological differentiation of HCC correlates with the decreases in the level and activity of HNF-1α. In a mouse model, loss of HNF4 expression is an important determinant of HCC invasion and progression which were further confirmed in a resistant hepatocyte (RH) male rat model [22]. Cereghini et al. in 1990 proved that HNF-1 transcripts are present only in differentiated hepatoma cells and no transcripts were detected in dedifferentiated variants [23]. HNF-1 along with LRH-1/hB1F synergistically enhances DNA replication. Subsequent studies revealed that a short region between − 118 and − 8 of HNF-1 promoter is vital for the cell type-specific expression of HNF-1 in hepatoma cells and HepG2 cells [24].

### Attempts to unravel the underlined molecular mechanisms

With the greater realization of HNF-1α-inactivated hepatic adenomas and HCCs, the scientific temptation to find its signaling transduction mechanism has become a natural drive. HNF-1α-regulated lipid and glucose metabolisms face greater chances of metabolic repercussions in the backdrop of liver cancers. One of the attempts is made by a group of Chinese researchers in 2015, which implicated TNF-α-induced inhibition of HNF-1α downstream that promoted HCC disease progress. Interestingly, a parallel suppression of liver-enriched miR-194 was also found, affected by TNF-α. With evidences, authors proposed that TNF-α/NF-κB pro-HCC signaling is achieved at least partly by the suppression of HNF-1α [25]. The mechanism is further elucidated by other groups recently. For example, Ding et al. revealed that HNF-1α regulated the expression of HNF1A-AS1 in HCC cells. HNF1A-AS1 in turn blunted the metastasis and tumorigenesis by directly binding to the C-terminal of SHP-1. Interestingly, promotion effects of HNF-1α and HNF1A-AS1 were reversed when SHP-1 enzymatic activity was inhibited, demonstrating that HNF-1α/HNF1A-AS1/SHP-1 axis is involved in the anti-HCC actions and presents a potential treatment option [26]. However, others reported the tumor-promoting role of HNF1A-AS1 in HCC. The detailed role of HNF1A-AS1 in HCC should be further clarified.

**Fig. 1:**
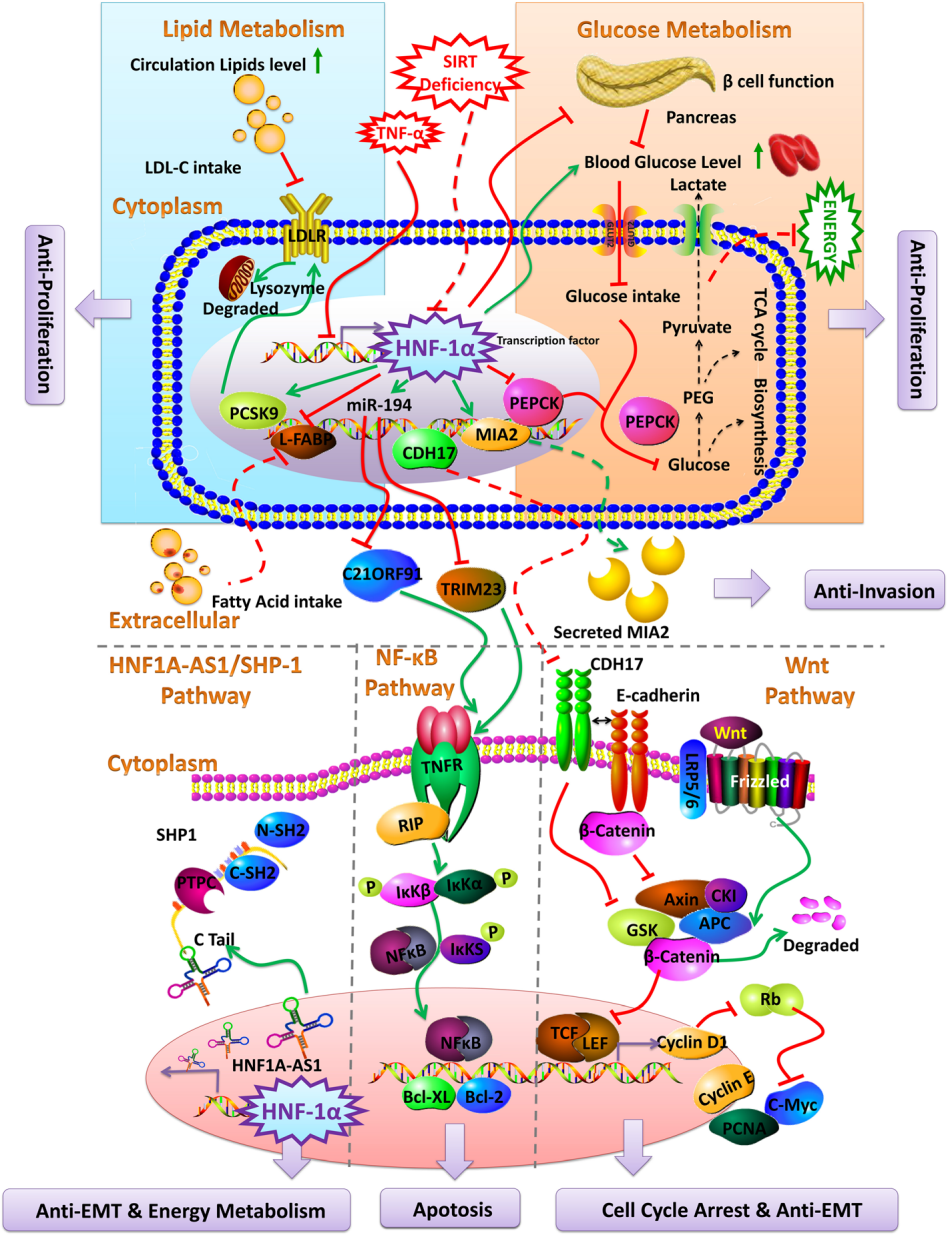
Schematically outlines the reported metabolic background of HNF-1α regulatory mechanism networks in various types of liver malignancies. Blockade of glucose energy metabolism by HNF-1α-driven signaling at different steps shunts the required energy that results in initiation of anti-proliferative mechanisms. On the other hand, HNF-1α also affects lipid metabolism by activating PCSK9 and inhibiting L-FABP. PCSK9 eventually leads to lysosomal degradation resulting in activation of anti-proliferative mechanisms. In the sidelines of metabolic repercussion, TNF-α blocks HNF-1α signaling which removes the NF-ƙβ signaling blockade, ultimately giving rise to malignant manifestations in the liver. Independently, HNF-1α activates HNF1A-AS1/SHP-1 signaling that has an alleviating effect on different liver malignancies. Similarly, MIA-2 is activated by HNF-1α that has showed to curtail tumorigenesis in different hepatic cancer models. A relatively well-known pro-tumorigenic cadherin-17 is documented to be blocked by HNF-1α that activates Wnt signaling, resulting in anti-carcinogenic effects within liver. SIRT is an intracellular metabolic regulator, whose deficiency is reported to inhibit HNF-1α eventually leading to carcinogenic signaling

Hepassocin (HPS) expressed predominantly in the liver. Expression of the HPS/LFIRE-1 (liver fibrinogen-related gene-1) is widely reported to be downregulated or lost completely in HCC tissues compared with their adjacent normal liver tissues, and their expression level was strongly associated with the tumor differentiation status [27]. Interestingly, deletion of the HNF-1 binding site not only led to a complete loss of HPS promoter activity, but also curtailed the induction of the HPS promoter by HNF-1α. Re-expression of HNF-1α in human hepatoma HepG2 cells re-induced HPS expression, whereas HNF-1α knockdown resulted in a notable decrease of HPS expression. It concludes that the HNF-1 binding site and HNF-1α are critical to liver-specific expression of HPS. SIRT1 is a member of sirtuins, and it is an important regulator for a variety of cellular processes, spanning from energy metabolism, stress response, to tumorigenesis and aging. Interestingly, hepatic deficiency of SIRT1 leads to decreased HNF-1α/FXR signaling activity, which reduces hepatic bile acid excretion and raises the chances of liver damage [28]. One of the important attempts to evaluate the tumor-suppressive role of HNF-1 is achieved a decade ago. The expression of melanoma inhibitory activity-2 (MIA-2), a tumor suppressor, is positively regulated by HNF-1. Loss of HNF-1 resulted in MIA-2 downregulation which culminates in HCC. Re-expression of HNF-1 and MIA-2 resulted in significantly slower and less invasive growth pattern of HCC [29]. Mice lacking HNF-1 gene failed to thrive and died after a progressive wasting syndrome with notable hepatic enlargement. It was further learned that the mice had higher plasma transaminases, cholesterol, mild hyperbilirubinemia, mild hyperammonemia, very high hydroxyproline levels, and severe hyperphenylalaninemia. Inorganic arsenic is a source of hepatic carcinogenesis. Interestingly, HNF-1α and HNF-4α are downregulated in inorganic arsenic-induced hepatic cancers. Cadherin-17 (CDH17) is aberrantly expressed in HCC and appears to be an attractive therapeutic target for liver malignancies [30]. HNF-1α and CDX2 could transcriptionally activate the expression of CDH17 by binding to its promoter in HCC cells. Decreased expression of CDH17, HNF-1α, and CDX2 was found in the liver of mouse during development, as well as in human HCC cells with less metastatic potential. This implies the diverse effects of HNF-1α in liver malignancies. Tumor-promoting actions of CDH17 were further dilated by Liu et al. that held inhibition of Wnt signaling responsible for anti-tumor effects, once CDH17 is blunted [31]. This may casually explain the relationship between CDH17 and HNFs. An interesting observation is shared by Balabaud et al., suggesting the abundance of H-HCAs as “micro and small HCAs cannot all be detected by routine ultrasound” [32]. Although greater majority of scientific data implicates HNF-1α to HCCs or HCAs, there is a contradictory finding, which may hint towards species-specific behavior of HNF-1α as conflicting study was reported in other than human species. Grace et al. failed to identify mutations in HNF-1α genes in tumors that were induced by DEN+CCl_4_ administration in B6C3F1 mice [33]. Together, these data suggest that HNF-1α regulates liver malignancies through various types of metabolic pathway (Fig. 1).

### HNF-1α impacts on stem cells

Cellular differentiation is a basic process that partly depends upon transcription factors and co-factors [34, 35]. Stem cells play a vital role in cellular differentiation and affect the normal and malignant cellular fate [36]. Recent advances revealed a potential role of HNF-1α in stemness. Griscelli et al. generated induced pluripotent stem cells (iPSCs) from a patient with HNF1A p.S142F mutation that had normal karyotype, harbored the HNF1A p.S142F mutation, and expressed it as pluripotency hallmarks [37]. Wharton’s jelly-mesenchymal stem cells (WJ-MSCs) have potential to develop into hepatocytes with HNF-1α as one of its markers [38], suggesting that HNF-1α may play a role in stem cell differentiation. However, Godoy et al. maintained that HNF-1 controlled hepatocyte-like cells that were differentiated from stem cells “strongly overlaps with genes repressed in cultivated hepatocytes,” slamming the current in vitro cultivating technique to be unable to help stem cells develop into hepatocytes [39].

**Fig. 2:**
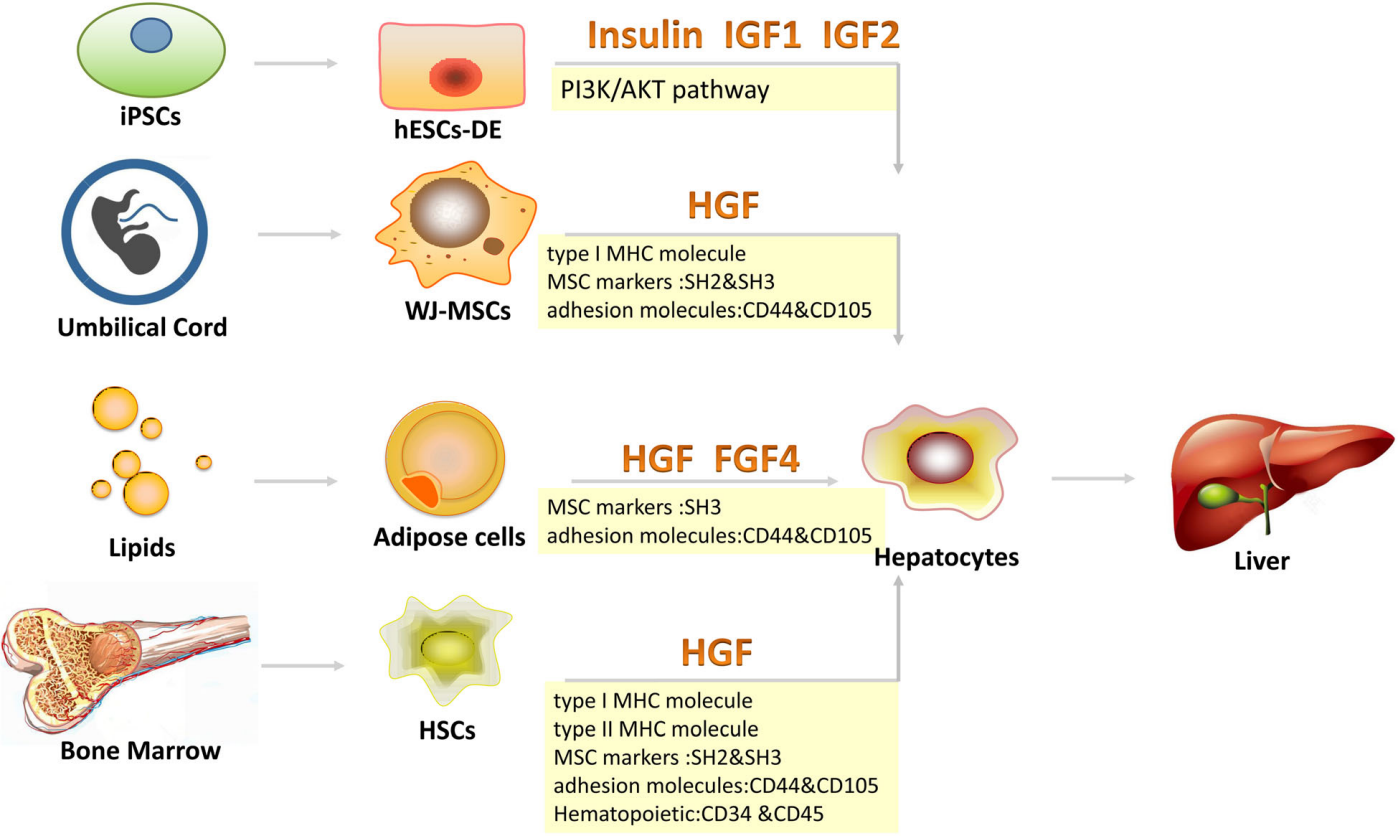
Effects of HNF-1α on different stem cells conversion into the liver. Various scientific reports have concluded that HNF-1α impacts iPSC, WJ-MSC, adipose mesenchymal stem cells (AMSCs), and HSC for their maturation into hepatocytes and hence the liver as an organ. The understanding of relative control of HNF-1α in liver formation is vital to comprehend its role in liver malignancies. It is, however, unknown what role HNF-1α plays in liver malignancies by affecting stem cells and remains an interesting area to unravel

Furthermore, visceral endoderm in the yolk sac expressed HNF1 at around 8.5th embryonic day while pancreatic and liver cells manifested it at 10.5th day [40]. In addition, Pontoglio et al. found that inactivation of HNF-1 at early stages of life can result in hepatic dysfunction and HNF-1-deficient mice died during postnatal life [41]. In 2013, Magner et al. showed insulin as a factor in hepatocyte differentiation from hESC-derived definitive endoderm (DE) and revealed that the PI3K pathway regulates hepatocyte differentiation from DE by increasing HNF1 and HNF4 expression [42]. Swenson and companions maintained that the use of a nuclear marker (HNF1) of mature hepatocytes or cholangiocytes boosts the differentiation of marrow-derived epithelial cells in tissues [43]. One of the earliest scientific validation of HNF1 role in stem cell growth and differentiation was put forward by Haumaitre et al., implicating the key roles of HNF1 family members in differentiation of the visceral endoderm cell lineage [44]. In continuation, Nagy et al. described that established transcription factors like HNF-1 and HNF-3 provide an impetus for oval cell activation [34], which ultimately differentiate into liver cells. Moreover, Barone and companions injected bone marrow stem cells (BMSCs) from healthy wild-type male mice into 14-month-old female mice having apparently HCC. It was derived that Y-chromosome/HNF1-positive cells in the liver validate the existence of a cell fusion process but BMSCs do not take part in carcinogenesis process [45]. The presence of HNF1-positive cells without any participation in carcinogenesis process corresponds with its anti-tumor actions discussed in the previous sections, though recent emergence of HNF1α as a novel oncogene and a central regulator of pancreatic cancer stem cells [46] presents a potentially interesting antithesis with respect to above facts but remains alone for instance. It yet remains to establish what kind of impacts does HNF-1α exert on cancerous cells owing to its differentiating relationship with stem cells, but it also presents a future opportunity to explore a possible and potential breakthrough (Fig. 2).

In conclusion, liver malignancies and hepatic metabolic dysfunctioning are both interconnected in various ways and cell stemness can affect both states. Recent evidences have proved that liver metabolic diseases, including manifestation of diabetes and metabolic syndrome, are affected by complex gene interactions. For example, human iPSCs are extracted from both type 1 and type 2 diabetes patients [47]. Since MODY3 diabetes is also a monogenic disease with more than 200 *HNF1A* mutations, its effects on cell stemness may provide the basis for the mutations and their phenotypic manifestation. Using a human embryonic stem cell model, Cardenas-Diaz et al. reported that HNF-1α was required to suppress an alpha cell gene expression, and then regulate endocrine cell function and cellular metabolism [48].

There is a scarcity of scientific reports targeting HNF-1α cell stemness with perspective of studying metabolic consequences, and hence, this subject overall remains obscure, but it has a great potential and can serve as one of the targets. Efficient scientific modeling remains one of the key problems to study cell stemness. One of the solutions, provided by Teo et al., is to isolate hiPSCs from metabolic dysfunctioning patients and then differentiate them and transplant into immunocompromised mice for in vivo maturation [49]. One such example is lesser glucose sensitivity showed by hiPSC-derived β-cells in MODY 2 model with *GSK* mutations. In addition, Kazuo Takayama et al. found that transduction of HNF-1α represents a valuable apparatus for the effective production of metabolically functional hepatocytes from hESCs and hiPSCs [50] that can serve as potential model for future research.

## Conclusion

HNF-1α is a transcription factor having considerable role in liver malignancies and cell stemness with metabolic repercussions. Abundant clinical and laboratory data concluded that downregulation of HNF-1α correlated with the progression in liver malignancies that persist with metabolic dysregulations. MODY3 patients specifically harboring HNF-1α mutations are more prone to develop malignancies along with metabolic insufficiencies than those with wild-type HNF-1α. Furthermore, HNF-1α is linked with cell stemness, established as one of the markers of hepatocytes developed from various stem cells. Conversely, stem cells lacking HNF-1α expression could not develop into normal hepatocytes, presenting a viable opportunity for malignancies and metabolic dysregulations. The exact mechanism through which HNF-1α acts remains obscure, but TNF-α, SHP-1, CDH17, SIRT, and MIA-2 are reported in HNF-1α regulations to date. The data on HNF-1α and its impacts on cell stemness and liver malignancies are still in the growing phase. This subject presents a novel futuristic opportunity to harness HNF-1α as a target to understand and treat liver malignancies, possibly by impacting cell stemness.

## Data Availability

Not applicable.

## Abbreviations

AMSCs: Adipose mesenchymal stem cells
CDH-17: Cadherin-17
DE: Definitive endoderm
EMT: Epithelial-mesenchymal transition
HA: Hepatocellular adenoma
HNF-1α: Hepatocyte nuclear factor-1 alpha
HPS: Hepassocin
iPSCs: Induced pluripotent stem cells
MIA-2: Melanoma inhibitory activity-2
NF-ƙβ: Nuclear factor
OCC: Ovarian clear cell carcinoma
PCSCs: Pancreatic cancer stem cells
RCC: Renal cell carcinoma
SHP-1: Src homology region 2 domain-containing phosphatase-1
SIRT: Sirtuin
TNF-α: Tumor necrosis factor-α
WJ-MSCs: Wharton’s jelly-mesenchymal stem cells
